# Real-world efficacy and safety of anlotinib in Chinese solid tumors patients with liver metastases: a multicenter retrospective study

**DOI:** 10.3389/fphar.2026.1639934

**Published:** 2026-05-15

**Authors:** Yanru Qin, Yujia Zhu, Minglei Zhuo, Xiaoxian Xu, Zhehai Wang

**Affiliations:** 1 Department of Oncology, The First Affiliated Hospital of Zhengzhou University, Zhengzhou, China; 2 Department of Radiation Oncology, Sun Yat-Sen University Cancer Center, Guangzhou, China; 3 Department I of Thoracic Oncology, Peking University Cancer Hospital and Institute, Beijing, China; 4 Department of Abdominal Radiotherapy, Cancer Hospital of the University of Chinese Academy of Sciences/Zhejiang Cancer Hospital, Hangzhou, China; 5 Department of Medical Oncology, Cancer Hospital of Shandong First Medical University (Shandong Cancer Institute, Shandong Cancer Hospital), Jinan, China

**Keywords:** anlotinib, liver metastases, real-world data, retrospective, clinical benefits, manageable safety

## Abstract

**Introduction:**

Liver metastases are associated with dismal prognosis in solid tumors. This retrospective study evaluated the efficacy and safety of anlotinib in patients with solid tumors and liver metastases in the real-world setting.

**Methods:**

Patient data from 5 centers in China were reviewed, and patients treated with anlotinib-based therapy were included in a single group. Tumor response was evaluated according to the Response Evaluation Criteria in Solid Tumors version 1.1. The primary outcomes were objective response rate (ORR) and progression-free survival (PFS). Secondary outcomes included hepatic ORR (hORR), hepatic PFS (hPFS), overall survival (OS), duration of response (DOR), and safety.

**Results:**

Between January 2020 and February 2023, 475 patients were included. Median PFS was 5.80 months (95% confidence interval [CI]: 5.27-6.60). The ORR and hORR were 17.89% and 18.74%, with a median DOR of 8.63 months (95% CI: 6.57 to not reached [NR]) and 8.80 months (95% CI: 7.51-NR), respectively. Median hPFS and OS were 6.03 months (95% CI: 5.33-6.60) and 9.53 months (95% CI: 8.40-NR), respectively. Similar median PFS, hPFS, and OS were observed across different tumor types. Patients with body mass index (BMI) <18.5 kg/m^2^ had significantly shorter median PFS and hPFS than those with BMI of 18.5-23.9 kg/m^2^ and ≥24.0 kg/m^2^. The incidence of any-grade adverse events was 19.58%, with the most common being hematological toxicities (14.11%). Grade ≥3 adverse events occurred in 1.68% of the patients.

**Discussion:**

These findings suggest the clinical benefits of anlotinib for the treatment of solid tumors with liver metastases, with a manageable safety profile.

## Introduction

The liver is one of the most common organs for metastases from solid tumors of non-hepatic origin, especially colorectal cancer, pancreatic cancer, and lung cancer (Horn et al., 2020; Wang et al., 2021). Liver metastases can negatively affect patient prognosis. The median overall survival (OS) was only 1–11 months in patients with solid tumors and synchronous liver metastases (Horn et al., 2020; Wang et al., 2021). The efficacy of current treatment regimens (including surgery, locoregional therapy, and systemic therapy) remains limited, suggesting an urgent need for novel effective therapies for the treatment of liver metastases.

Angiogenesis is an important phase during the process of liver metastases, and delivers oxygen and nutrients to cancer cells. One of the mechanisms of angiogenesis is the recruitment of neutrophils, which induces the secretion of fibroblast growth factor (FGF) 2, stimulating vascular remodeling and formation of more functional vessels (Gordon-Weeks et al., 2017). Another mechanism of vascular co-option allows metastatic cells to survive through obtaining oxygen and nutrients from existing vessels (Frentzas et al., 2016). In addition, liver metastases can siphon antigen-specific CD8^+^ T cells and induce systemic immunosuppression, leading to diminished response to immunotherapy (Yu et al., 2021).

Antolinib is an oral tyrosine kinase inhibitor that targets vascular endothelial growth factor receptors (VEGFR) one to three, platelet-derived growth factor receptors (PDGFR) α and β, fibroblast growth factor receptors (FGFR) 1-4, and c-Kit (Sun et al., 2016; Shen et al., 2018). It can induce durable tumor vascular normalization (Fan et al., 2022), suppress tumor growth (Wang et al., 2019), improve hypoxia (Yang L. et al., 2020), and enhance radiosensitivity (Han et al., 2022). It can also downregulate programmed cell death-ligand 1 (PD-L1) expression (Liu et al., 2020), increase the infiltration of immune cells (Liu et al., 2020; Luo et al., 2024; Yang Y. et al., 2020; Fan et al., 2022; Song et al., 2024), improve the ratio of CD8^+^ T cells to FoxP3^+^ T cells (Liu et al., 2020), and potentiate the antitumor effect of immunotherapy. In China, anlotinib has been approved for the treatment of advanced lung cancer (Han et al., 2018; Cheng et al., 2021; Cheng et al., 2024), soft tissue sarcoma (Chi et al., 2018), and thyroid carcinoma (Li et al., 2021; Chi et al., 2023). The role of anlotinib against liver metastases has also been explored in several clinical trials (Cheng et al., 2022; Shen et al., 2023; Qiu et al., 2024), but the sample size was limited. Whether anlotinib can provide comparable intrahepatic and extrahepatic control still needs further investigations. In addition, large-scale real-world evidence on the effect of anlotinib in the treatment of liver metastases is lacking.

This study aimed to evaluate the efficacy and safety of anlotinib in patients with solid tumors and liver metastases in a real-world setting.

## Materials and methods

### Study design

This multicenter retrospective study was conducted at five centers in China. Patients who visited the hospital between 1 January 2020 and 28 February 2023 were searched through the hospital information system. This study was approved by the ethics committee of each participating center. The requirement for informed consent was waived owing to the retrospective nature of the study.

### Patients

The inclusion criteria were as follows: (1) documented clinical diagnosis of solid tumors with liver metastases; (2) records of continuous anlotinib treatment for at least 2 weeks during the treatment period; and (3) at least one imaging examination before anlotinib treatment and at least two imaging examinations after the initiation of anlotinib treatment.

The exclusion criteria were: (1) documented diagnosis of primary liver cancer; or (2) application of stereotactic body radiotherapy, stereotactic ablative radiotherapy, whole-brain radiotherapy, or transarterial chemoembolization (TACE) during anlotinib treatment.

### Treatment and assessments

Anlotinib was administered on days 1–14 of each 21-day cycle until disease progression or intolerable toxicity. Physicians decided the starting dose of anlotinib (8–12 mg) and treatment regimen (anlotinib monotherapy or in combination with other therapies) based on patient condition, and could adjust the dose for the management of adverse events (AEs).

Baseline characteristics were extracted from medical records, including sex, age, height, weight, tumor type, duration of liver metastases, number of liver metastases, treatment line of anlotinib in advanced settings, and combined drugs. Non-mandatory imaging examinations were conducted every 6 weeks. The report and date of each imaging examination and the living status of the patients were collected locally at each center to evaluate treatment efficacy. Tumor response at the primary tumor and liver metastases and systemic response were evaluated according to the Response Evaluation Criteria In Solid Tumors version 1.1. AEs that occurred during anlotinib treatment and dose reduction, interruption, or discontinuation of anlotinib due to AEs were collected to evaluate safety. AEs were graded according to the National Cancer Institute Common Terminology Criteria for Adverse Events, version 5.0.

## Outcomes

The primary outcomes were objective response rate (ORR) and progression-free survival (PFS). ORR was defined as the proportion of patients with the best systemic response of complete response (CR) or partial response (PR). PFS was defined as the time from the initiation of anlotinib treatment to progression at any site or death from any cause, whichever occurred first.

The secondary outcomes included hepatic ORR (hORR; defined as the proportion of patients with the best intrahepatic response of CR or PR), hepatic PFS (hPFS; defined as the time from the initiation of anlotinib treatment to intrahepatic progression or death from any cause, whichever occurred first), OS (defined as the time from anlotinib treatment initiation to death from any cause), duration of response (DOR; defined as the time from CR or PR to progression at any site or death from any cause, whichever occurred first), and safety.

### Statistical analysis

Continuous variables were expressed as mean ± standard deviation or median (range), while categorical variables were expressed as frequency and percentage. PFS, hPFS, OS, and DOR were estimated using the Kaplan-Meier method, and their 95% confidence intervals (CIs) were calculated using the Brookmeyer-Crowley method. Follow-up duration was estimated using the reverse Kaplan-Meier method. PFS and hPFS were not compared statistically. Subgroup comparisons were performed according to age, sex, body mass index (BMI), tumor type, duration of liver metastases, and treatment regimen using the log-rank test for PFS, hPFS, and OS. All the statistical analyses were performed using the R studio and Python. Two-sided P < 0.05 was considered statistically significant.

## Results

### Patient characteristics

**FIGURE 1:**
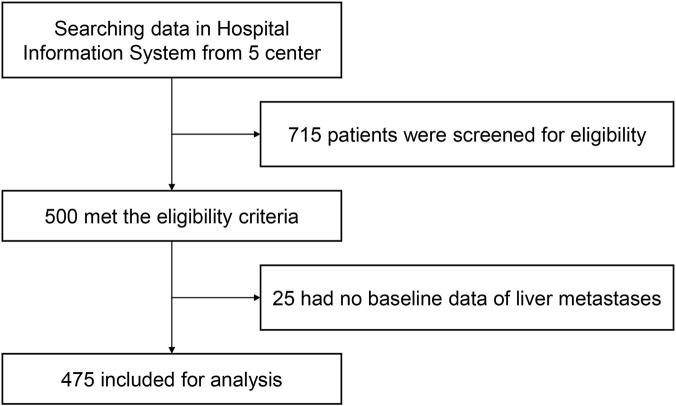
Patient flowchart.

A total of 715 patients were screened, and 500 of them met the eligibility criteria. While 25 patients had no baseline data of liver metastases, 475 patients were finally included in the analysis (Figure 1). The mean age was 65.5 ± 10.62 years. The majority of the patients were male (67.37%), had liver metastases for at least 12 months (75.16%), had multiple liver metastases (95.16%), received anlotinib as third-line treatment in the advanced setting (90.53%), and received anlotinib monotherapy (83.16%). The starting dose of anlotinib was 12 mg/day in almost all patients (97.26%). Forty (8.42%) patients received anlotinib plus a programmed cell death-1 (PD-1) inhibitor (including sintilimab [n = 22], camrelizumab [n = 13], and toripalimab [n = 5]), and 40 (8.42%) patients received anlotinib plus chemotherapy (including carboplatin [n = 12], capecitabine and oxaliplatin [n = 8], pemetrexed [n = 6], etoposide [n = 5], paclitaxel [n = 3], cisplatin and fluorouracil [n = 3], etoposide and cisplatin [n = 1], docetaxel [n = 1], and S-1 [n = 1]). The main tumor types included in this study were non-small-cell lung cancer (NSCLC; 20.63%), gastric cancer (15.37%), colorectal cancer (14.95%), and breast cancer (10.11%; Table 1). Baseline characteristics in patients with different tumor types are shown in Supplementary Table S1. All patients with gynecological cancers received anlotinib-based therapy as first-line treatment in the advanced setting, while at least 70% of patients with other cancers received third-line anlotinib-based treatment. Patients with breast cancer mostly received anlotinib plus a PD-1 inhibitor or chemotherapy (77.08%), while at least 85% of patients with other cancers received anlotinib monotherapy. By the data cutoff date on 28 February 2023, the median duration of follow-up was 6.67 months (95% CI: 6.40–6.97). Median duration of treatment was 4.14 months (range, 0.69–9.36) in the total population, 4.17 months (range, 0.69–9.36) in patients with lung cancer, 4.12 months (range, 0.69–8.71) in gastrointestinal cancer, 4.39 months (range, 0.69–7.43) in gynecological cancer, and 4.11 months (range, 1.54–7.62) in breast cancer.

**TABLE 1:** Baseline characteristics.

Characteristics	Patients (n = 475)
Age (years), mean ± SD	65.5 ± 10.62
<49	46 (9.68)
50–64	157 (33.05)
65–74	164 (34.53)
≥75	108 (22.74)
Sex, n (%)	
Male	320 (67.37)
Female	155 (32.63)
BMI (kg/m^2^), mean ± SD	20.9 ± 2.89
<18.5	104 (21.89)
18.5–23.9	300 (63.16)
24.0–28.0	71 (14.95)
Tumor type, n (%)	
Non-small cell lung cancer	98 (20.63)
Gastric cancer	73 (15.37)
Colorectal cancer	71 (14.95)
Breast cancer	48 (10.11)
Esophagus cancer	46 (9.68)
Small cell lung cancer	43 (9.05)
Ovarian cancer	25 (5.26)
Biliary tract cancer	24 (5.05)
Pancreatic cancer	24 (5.05)
Cervical cancer	23 (4.84)
Duration of liver metastases (months), n (%)	
<6	34 (7.16)
6–11	84 (17.68)
12–23	166 (34.95)
≥24	191 (40.21)
Number of liver metastases, n (%)	
1	23 (4.84)
≥2	452 (95.16)
Treatment line of anlotinib in advanced setting, n (%)	
1	9 (1.89)
2	36 (7.58)
3	430 (90.53)
Starting dose of anlotinib (mg/day), n (%)	
8	4 (0.84)
10	9 (1.89)
12	462 (97.26)
Treatment regimen, n (%)	
Anlotinib alone	395 (83.16)
Anlotinib plus PD-1 inhibitor	40 (8.42)
Anlotinib plus chemotherapy	40 (8.42)

SD, standard deviation; BMI, body mass index; PD-1, programmed cell death-1.

### Survival analysis

**FIGURE 2:**
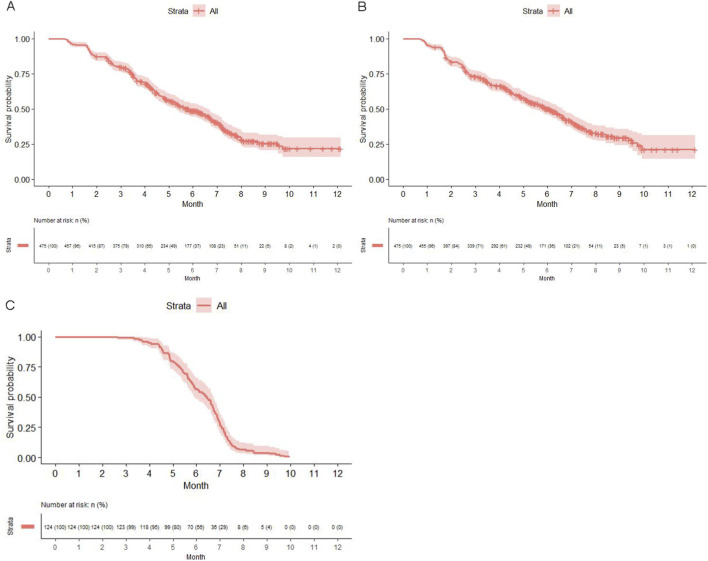
Kaplan-Meier curves for progression-free survival **(A)**, hepatic progression-free survival **(B)**, and overall survival **(C)**.

Median PFS was 5.80 months (95% CI: 5.27–6.60) in the total population (Figure 2A), with 287 (60.42%) PFS events. No significant difference in median PFS was observed in subgroups by age, sex, tumor type, or treatment regimen (anlotinib monotherapy, anlotinib plus a PD-1 inhibitor, or anlotinib plus chemotherapy). Median PFS was 7.70 months (95% CI: 5.43 to not reached [NR]) in patients with biliary tract cancer, 6.70 months (95% CI: 4.57–7.60) in cervical cancer, 6.60 months (95% CI: 4.00–NR) in pancreatic cancer, 6.43 months (95% CI: 4.89–7.23) in colorectal cancer, 6.30 months (95% CI: 4.93–NR) in breast cancer, 6.30 months (95% CI: 3.57–NR) in ovarian cancer, 5.80 months (95% CI: 5.00–7.20) in NSCLC, 5.63 months (95% CI: 4.57–7.60) in esophagus cancer, 5.43 months (95% CI: 4.30–NR) in small cell lung cancer, and 4.87 months (95% CI: 4.40–7.30) in gastric cancer. Patients with BMI <18.5 kg/m^2^ (4.57 months) had significantly shorter median PFS than those with BMI of 18.5–23.9 kg/m^2^ (6.60 months) and ≥24.0 kg/m^2^ (5.90 months, P = 0.001). In the subgroups by duration of liver metastases, patients with liver metastases for <6 months had the longest median PFS (8.10 months; Supplementary Table S2).

Median hPFS was 6.03 months (95% CI: 5.33–6.60) in the total population (Figure 2B), with 273 (57.47%) hPFS events. No significant difference in median hPFS was observed in subgroups by age, tumor type, or treatment regimen. Median hPFS was 7.27 months (95% CI: 4.53–NR) in patients with small cell lung cancer, 6.43 months (95% CI: 5.00–NR) in NSCLC, 6.37 months (95% CI: 4.53–NR) in pancreatic cancer, 6.20 months (95% CI: 4.60–NR) in gastric cancer, 6.13 months (95% CI: 5.17–7.60) in colorectal cancer, 5.90 months (95% CI: 3.47–NR) in ovarian cancer, 5.63 months (95% CI: 4.50–7.50) in esophagus cancer, 5.60 months (95% CI: 3.17–NR) in biliary tract cancer, 5.07 months (95% CI: 2.73–NR) in cervical cancer, and 4.83 months (95% CI: 3.20–6.60) in breast cancer. Male patients had a significantly longer median hPFS than female patients (6.47 months vs. 4.90 months, P = 0.012). Patients with BMI < 18.5 kg/m^2^ (4.53 months) had significantly shorter median hPFS than those with BMI of 18.5–23.9 kg/m^2^ (6.50 months) and ≥24.0 kg/m^2^ (6.93 months, P < 0.001). In the subgroups by duration of liver metastases, patients with liver metastases for <6 months had the longest median hPFS (7.63 months; Supplementary Table S3).

Median OS was 9.53 months (95% CI: 8.40–NR) in the total population (Figure 2C), with 124 (26.11%) deaths. No significant difference in median OS was observed across different subgroups. Median OS was NR (95% CI: 7.70–NR) in patients with biliary tract cancer, NR (95% CI, 7.37–NR) in small cell lung cancer, NR (95% CI, 7.17–NR) in breast cancer, NR (95% CI: 6.73–NR) in pancreatic cancer, NR (95% CI: 6.67–NR) in cervical cancer, 9.93 months (95% CI: 9.93–NR) in gastric cancer, 9.10 months (95% CI: 7.40–NR) in colorectal cancer, 8.43 months (95% CI: 6.83–NR) in esophagus cancer, 7.63 months (95% CI: 7.20–NR) in NSCLC, and 7.33 months (95% CI: 6.87–NR) in ovarian cancer (Supplementary Table S4).

### Tumor response

In the total population, the ORR and hORR were 17.89% and 18.74%, with a median DOR of 8.63 months (95% CI: 6.57–NR) and 8.80 months (95% CI: 7.51–NR; Table 2), respectively. The highest ORR and hORR were observed in patients with gastrointestinal cancer (21.85% and 20.17%), followed by lung cancer (15.60% and 19.15%), breast cancer (12.50% and 18.75%), and gynecological cancer (10.42% and 10.42%). Median DOR was similar across different tumor types. Median duration of systemic response and DOR at liver metastases were NR (NR–NR) and NR (NR–NR) in patients with gynecological cancer, and 8.63 months (6.03–NR) and 8.80 (6.80–NR) in gastrointestinal cancer (Supplementary Table S5).

**TABLE 2:** Tumor response by response evaluation criteria in solid tumors version 1.1.

Response	Primary tumor (n = 475)	Liver metastases (n = 475)	Systemic response (n = 475)
Best response, n (%)			
Partial response	77 (16.21)	89 (18.74)	85 (17.89)
Stable disease	377 (79.37)	346 (72.84)	384 (80.84)
Progressive disease	21 (4.42)	40 (8.64)	6 (1.26)
ORR, (%)	77 (16.21)	89 (18.74)	85 (17.89)
DOR (months), median (95% CI)	8.63 (6.57–NR)	8.80 (7.51–NR)	8.63 (6.57–NR)

ORR, objective response rate; DOR, duration of response; CI, confidence interval; NR, not reached.

### Safety

Of the 475 patients, the incidence of any grade AEs was 19.58%, with the most common being hematological toxicities (14.11%). All grade ≥3 AEs were hematological toxicities, which occurred in 1.68% of patients. Seventeen (3.58%) patients experienced dose reduction or interruption of anlotinib owing to AEs. Three patients (0.63%) discontinued anlotinib owing to AEs. No AEs resulted in death (Table 3). The incidence of any-grade AEs and hematological toxicities was the lowest in patients with gastrointestinal cancer (12.61% and 9.66%), followed by breast cancer (22.92% and 14.58%), lung cancer (26.95% and 19.15%), and gynecological cancer (29.17% and 20.83%; Supplementary Table S6).

**TABLE 3:** AEs.

Event, n (%)	Patients (n = 475)	
	Any grade	Grade ≥ 3
Any AE	93 (19.58)	8 (1.68)
AE leading to dose reduction or interruption of anlotinib	17 (3.58)	0
AE leading to anlotinib discontinuation	3 (0.63)	1 (0.21)
AE leading to death	0	0
Hematological toxicities	67 (14.11)	8 (1.68)
Gastrointestinal toxicities	17 (3.58)	0
Pain	7 (1.47)	0
Fatigue	1 (0.21)	0

AE, adverse event.

## Discussion

To our knowledge, this is the first study to focus on the efficacy and safety of anlotinib in patients with solid tumors and liver metastases based on real-world data. The results showed a median PFS of 5.80 months, a median hPFS of 6.03 months, and a median OS of 9.53 months. Similar median PFS, hPFS, and OS were observed across different tumor types. The safety profile was acceptable, whether in the total population or in each tumor type.

In our study, more than half of the patients (57.26%) were aged ≥65 years, and almost all patients (95.16%) had multiple liver metastases. Anlotinib was mostly used as third-line treatment for advanced disease (90.53%), and monotherapy was the most common regimen (83.16%). In such circumstance, the median PFS and OS in this real-world study were similar to results (median PFS: 4.1–5.4 months; median OS: 7.3–9.6 months) from previous randomized controlled trials of third- or further-line anlotinib in lung cancer (Han et al., 2018; Cheng et al., 2021). Two previous clinical trials have explored bevacizumab plus chemotherapy in heavily pretreated patients with advanced solid tumors and liver metastases. The median OS was 7–8.6 months and the median PFS was 4.2 months (Tsimberidou et al., 2013a; Tsimberidou et al., 2013b), slightly worse than our data. All of these indirect comparisons reflect the promising value of anlotinib when used in clinical practice. Additionally, the median PFS and hPFS were numerically similar in our study. In the phase 3 ALTER 0303 study, anlotinib could provide PFS benefits in both the total population with advanced NSCLC and subgroup patients with liver metastases (Han et al., 2018; Shen et al., 2023). These results suggest that both intrahepatic and extrahepatic lesions can be effectively controlled with anlotinib in later-line patients.

The subgroup analysis indicated that anlotinib combined with a PD-1 inhibitor or chemotherapy did not appear to improve survival compared with anlotinib monotherapy. An observational study showed that the median PFS (7.6 months vs. 4.1 months) and OS (15.7 months vs. 9.2 months) were significantly improved in the TACE plus regorafenib group compared with the regorafenib alone group in patients with colorectal cancer and liver metastases who failed standard treatments (Cao et al., 2021). The key subgroup analysis of the phase 3 Impower150 study demonstrated that the addition of atezolizumab to bevacizumab plus chemotherapy might lead to better PFS (median, 8.2 months vs. 5.4 months) and OS (median, 13.2 months vs. 9.1 months) benefits (Reck et al., 2019; Nogami et al., 2022). Unfortunately, the corresponding data of the abovementioned treatment strategies were not collected from our study centers. Whether anlotinib combined with TACE, immunochemotherapy, or other potentially effective regimens could further improve the clinical outcomes for patients with liver metastases need further investigations.

We also found that patients with BMI below the normal range experienced faster systemic and intrahepatic progression. This might be due to the fact that such patients had more aggressive tumors and showed evident weight loss, which in turn lead to worse nutritional status, insufficient muscle mass, and reduced daily activities and exercise, affecting prognosis (Martin et al., 2013; Bullock et al., 2020; Kiss et al., 2023). This finding indicate that more effective treatment regimens are warranted for patients with liver metastases and low BMI.

The incidence of AEs (19.58%) and dose adjustment rate of anlotinib due AE (4.21%) were low in our study, revealing good tolerance to anlotinib in clinical practice. However, owing to the retrospective and non-interventional design, monitoring and recording of AEs might be less rigorous and accurate than clinical trials. Missing reports of AEs might also exist in this real-world study. This may be supported by comparisons with previous clinical trial data (Han et al., 2018; Cheng et al., 2021; Chi et al., 2018; Li et al., 2021; Chi et al., 2023). The incidence of AEs in this study might have been underestimated and should be interpreted with caution.

The study had some limitations. First, potential selection and reporting biases were inevitable owing to the retrospective nature of the study, such as the possibly incomplete AE reporting. Second, only patients treated with anlotinib were included, without direct comparisons with other treatment regimens for liver metastases. Third, most patients did not have available data of Eastern Cooperative Oncology Group performance status or liver function. Fourth, this study included various tumor types and regimens, leading to high heterogeneity of the study population. The finding of similar efficacy across different tumor types may reflect the confounding effect of treatment line or disease biology rather than the universal activity of anlotinib. Fifth, imaging intervals were not standardized and evaluations were site-reported, which might introduce variability in PFS and hPFS estimations. Finally, the follow-up duration was short; thus, OS data were immature in many subgroups. Large-scale prospective studies are warranted to validate our findings.

## Conclusion

Anlotinib showed clinical benefits in patients with solid tumors and liver metastases, with a manageable safety profile. It may be used as an alternative later-line treatment option in these patients, warranting further validation.

## Data Availability

The original contributions presented in the study are included in the article/Supplementary Material, further inquiries can be directed to the corresponding author.
