# Supplementary material for: Real-world efficacy and safety of anlotinib in Chinese solid tumors patients with liver metastases: a multicenter retrospective study
Source: Front Pharmacol. 2026 May 15;17:1639934. doi: 10.3389/fphar.2026.1639934 (PMC13219273; doi:10.3389/fphar.2026.1639934)
Supplement: Supplementary file 1 [file Supplementaryfile1.docx]

**Supplementary Materials for**

**Real-world efficacy and safety of anlotinib in Chinese solid tumors patients with liver metastases: A multicenter retrospective study**

Yan-Ru Qin^1^, Yu-Jia Zhu^2^, Ming-Lei Zhuo^3^, Xiao-Xian Xu^4^, Zhe-Hai Wang^5^*

Correspondence to:

Zhe-Hai Wang: [wzhai8778@sina.com](mailto:wzhai8778@sina.com), wangzhehai2024@126.com

**This file includes:**

Supplementary Tables S1 to Tables S6

**Supplementary Table 1** Baseline characteristics in subgroups by tumor type

| **Characteristics** | **Lung cancer**  **(n=141)** | **Gastrointestinal cancer**  **(n=238)** | **Gynecological cancer**  **(n=48)** | **Breast cancer**  **(n=48)** |
| --- | --- | --- | --- | --- |
| Age (years), mean±SD |  |  |  |  |
| <65 | 57 (40.43) | 102 (42.86) | 21 (43.75) | 23 (47.92) |
| ≥65 | 84 (59.57) | 136 (57.14) | 27 (56.25) | 25 (52.08) |
| Sex, n (%) |  |  |  |  |
| Male | 124 (87.94) | 196 (82.35) | 0 | 0 |
| Female | 17 (12.06) | 42 (17.65) | 48 (100) | 48 (100) |
| BMI (kg/m^2^), mean±SD |  |  |  |  |
| <18.5 | 32 (22.70) | 53 (22.27) | 11 (22.92) | 8 (16.67) |
| 18.5-23.9 | 94 (66.67) | 152 (63.87) | 27 (56.25) | 27 (56.25) |
| 24.0-28.0 | 15 (10.64) | 33 (13.87) | 10 (20.83) | 13 (27.08) |
| Duration of liver metastases (months), n (%) |  |  |  |  |
| <6 | 16 (11.35) | 16 (6.72) | 0 | 2 (4.17) |
| 6-11 | 31 (21.99) | 33 (13.87) | 9 (18.75) | 11 (22.92) |
| 12-23 | 40 (28.37) | 92 (38.66) | 13 (27.08) | 21 (43.75) |
| ≥24 | 54 (38.30) | 97 (40.76) | 26 (54.17) | 14 (29.17) |
| Treatment line of anlotinib in advanced setting, n (%) |  |  |  |  |
| 1 | 0 | 2 (0.84) | 48 (100) | 7 (14.58) |
| 2 | 3 (2.13) | 27 (11.34) | 0 | 6 (12.50) |
| 3 | 138 (97.87) | 209 (87.82) | 0 | 35 (72.92) |
| Starting dose of anlotinib (mg/day), n (%) |  |  |  |  |
| 8 | 0 | 0 | 4 (8.33) | 0 |
| 10 | 5 (3.55) | 3 (1.26) | 1 (2.08) | 0 |
| 12 | 136 (96.45) | 235 (98.74) | 43 (89.58) | 48 (100) |
| Treatment regimen, n (%) |  |  |  |  |
| Anlotinib alone | 127 (90.07) | 215 (90.34) | 42 (87.50) | 11 (22.92) |
| Anlotinib plus PD-1 inhibitor | 8 (5.67) | 11 (4.62) | 5 (10.42) | 16 (33.33) |
| Anlotinib plus chemotherapy | 6 (4.26) | 12 (5.04) | 1 (2.08) | 21 (43.75) |

**Supplementary Table 2** Comparisons of PFS in subgroups

| **Subgroup** | **Number of patients** | **PFS (months), median (95% CI)** | **P** |
| --- | --- | --- | --- |
| Age (years) |  |  | 0.81 |
| <65 | 203 | 5.60 (5.00-6.73) |  |
| ≥65 | 272 | 5.83 (5.00-7.07) |  |
| Sex |  |  | 0.67 |
| Male | 320 | 6.10 (5.33-6.77) |  |
| Female | 155 | 5.30 (4.57-6.73) |  |
| BMI (kg/m^2^) |  |  | 0.001 |
| <18.5 | 104 | 4.57 (4.27-5.40) |  |
| 18.5-23.9 | 300 | 6.60 (5.63-7.17) |  |
| ≥24.0 | 71 | 5.90 (4.37-7.20) |  |
| Tumor type |  |  | 0.91 |
| Non-small cell lung cancer | 98 | 5.80 (5.00-7.20) |  |
| Small cell lung cancer | 43 | 5.43 (4.30-NR) |  |
| Gastric cancer | 73 | 4.87 (4.40-7.30) |  |
| Colorectal cancer | 71 | 6.43 (4.89-7.23) |  |
| Esophagus cancer | 46 | 5.63 (4.57-7.60) |  |
| Pancreatic cancer | 24 | 6.60 (4.00-NR) |  |
| Biliary tract cancer | 24 | 7.70 (5.43-NR) |  |
| Breast cancer | 48 | 6.30 (4.93-NR) |  |
| Ovarian cancer | 25 | 6.30 (3.57-NR) |  |
| Cervical cancer | 23 | 6.70 (5.27-NR) |  |
| Duration of liver metastases (months) |  |  | <0.001 |
| <6 | 34 | 8.10 (7.63-NR) |  |
| 6-11 | 84 | 5.40 (4.40-6.73) |  |
| 12-23 | 166 | 5.87 (4.87-7.27) |  |
| ≥24 | 191 | 5.10 (4.57-6.37) |  |
| Treatment regimen |  |  | 0.95 |
| Anlotinib alone | 395 | 5.80 (5.27-6.73) |  |
| Anlotinib plus PD-1 inhibitor | 40 | 5.53 (4.57-6.83) |  |
| Anlotinib plus chemotherapy | 40 | 6.13 (4.30-NR) |  |

PFS, progression-free survival; CI, confidence interval; BMI, body mass index; NR, not reached; PD-1, programmed cell death-1.

**Supplementary Table 3** Comparisons of hPFS in subgroups

| **Subgroup** | **Number of patients** | **HPFS (months), median (95% CI)** | **P** |
| --- | --- | --- | --- |
| Age (years) |  |  | 0.23 |
| <65 | 203 | 6.47 (5.60-6.93) |  |
| ≥65 | 272 | 5.70 (4.87-6.60) |  |
| Sex |  |  | 0.012 |
| Male | 320 | 6.47 (5.83-7.23) |  |
| Female | 155 | 4.90 (4.23-6.13) |  |
| BMI (kg/m^2^) |  |  | <0.001 |
| <18.5 | 104 | 4.53 (4.17-5.27) |  |
| 18.5-23.9 | 300 | 6.50 (5.70-7.17) |  |
| ≥24.0 | 71 | 6.93 (5.90-NR) |  |
| Tumor type |  |  | 0.76 |
| Non-small cell lung cancer | 98 | 6.43 (5.00-NR) |  |
| Small cell lung cancer | 43 | 7.27 (4.53-NR) |  |
| Gastric cancer | 73 | 6.20 (4.60-NR) |  |
| Colorectal cancer | 71 | 6.13 (5.17-7.60) |  |
| Esophagus cancer | 46 | 5.63 (4.50-7.50) |  |
| Pancreatic cancer | 24 | 6.37 (4.53-NR) |  |
| Biliary tract cancer | 24 | 5.60 (3.17-NR) |  |
| Breast cancer | 48 | 4.83 (3.20-6.60) |  |
| Ovarian cancer | 25 | 5.90 (3.47-NR) |  |
| Cervical cancer | 23 | 5.07 (2.73-NR) |  |
| Duration of liver metastases (months) |  |  | 0.027 |
| <6 | 34 | 7.63 (6.97-NR) |  |
| 6-11 | 84 | 6.03 (5.23-7.30) |  |
| 12-23 | 166 | 6.13 (4.90-7.50) |  |
| ≥24 | 191 | 5.20 (4.53 -6.43) |  |
| Treatment regimen |  |  | 0.48 |
| Anlotinib alone | 395 | 6.20 (5.43-6.77) |  |
| Anlotinib plus PD-1 inhibitor | 40 | 5.30 (4.53-6.67) |  |
| Anlotinib plus chemotherapy | 40 | 6.73 (4.90-NR) |  |

hPFS, hepatic progression-free survival; CI, confidence interval; BMI, body mass index; NR, not reached; PD-1, programmed cell death-1

**Supplementary Table 4** Comparisons of OS in subgroups

| **Subgroup** | **Number of patients** | **OS (months), median (95% CI)** | **P** |
| --- | --- | --- | --- |
| Age (years) |  |  | 0.50 |
| <65 | 203 | 8.43 (7.30-NR) |  |
| ≥65 | 272 | 9.53 (8.40-NR) |  |
| Sex |  |  | 0.96 |
| Male | 320 | 9.53 (7.83-NR) |  |
| Female | 155 | NR (7.43-NR) |  |
| BMI (kg/m^2^) |  |  | 0.13 |
| <18.5 | 104 | 7.43 (7.00-NR) |  |
| 18.5-23.9 | 300 | 9.73 (9.10-NR) |  |
| ≥24.0 | 71 | 7.50 (7.23-NR) |  |
| Tumor type |  |  | 0.92 |
| Non-small cell lung cancer | 98 | 7.63 (7.20-NR) |  |
| Small cell lung cancer | 43 | NR (7.37-NR) |  |
| Gastric cancer | 73 | 9.93 (9.93-NR) |  |
| Colorectal cancer | 71 | 9.10 (7.40-NR) |  |
| Esophagus cancer | 46 | 8.43 (6.83-NR) |  |
| Pancreatic cancer | 24 | NR (6.73-NR) |  |
| Biliary tract cancer | 24 | NR (7.70-NR) |  |
| Breast cancer | 48 | NR (7.17-NR) |  |
| Ovarian cancer | 25 | 7.33 (6.87-NR) |  |
| Cervical cancer | 23 | NR (6.67-NR) |  |
| Duration of liver metastases (months) |  |  | 0.14 |
| <6 | 34 | NR (8.10-NR) |  |
| 6-11 | 84 | 9.93 (7.47-NR) |  |
| 12-23 | 166 | 9.53 (8.40-NR) |  |
| ≥24 | 191 | 7.37 (7.17-NR) |  |
| Treatment regimen |  |  | 0.11 |
| Anlotinib alone | 395 | 9.53 (8.40-NR) |  |
| Anlotinib plus PD-1 inhibitor | 40 | 7.37 (6.67-NR) |  |
| Anlotinib plus chemotherapy | 40 | 7.40 (7.17-NR) |  |

OS, overall survival; CI, confidence interval; NR, not reached; BMI, body mass index; PD-1, programmed cell death-1

**Supplementary Table 5** Tumor response according to the Response Evaluation Criteria in Solid Tumors version 1.1 in subgroups by tumor type

| **Subgroup** | **Primary tumor** | **Liver metastases** | **Systemic response** |
| --- | --- | --- | --- |
| **Lung cancer** | n=141 | n=141 | n=141 |
| Best response, n (%) |  |  |  |
| Partial response | 21 (14.89) | 27 (19.15) | 22 (15.60) |
| Stable disease | 115 (81.56) | 106 (75.18) | 134 (95.04) |
| Progressive disease | 5 (3.55) | 8 (5.67) | 1 (0.71) |
| ORR, (%) | 21 (14.89) | 27 (19.15) | 22 (15.60) |
| DOR (months), median (95% CI) | 6.63 (6.63-NR) | NR (6.70-NR) | NR (NR-NR) |
| **Gastrointestinal cancer** | n=238 | n=238 | n=238 |
| Best response, n (%) |  |  |  |
| Partial response | 45 (18.91) | 48 (20.17) | 52 (21.85) |
| Stable disease | 179 (75.21) | 168 (70.59) | 222 (93.28) |
| Progressive disease | 14 (5.88) | 22 (9.24) | 5 (2.10) |
| ORR, (%) | 45 (18.91) | 48 (20.17) | 52 (21.85) |
| DOR (months), median (95% CI) | 8.63 (5.63-NR) | 8.80 (6.80-NR) | 8.63 (6.03-NR) |
| **Gynecological cancer** | n=48 | n=48 | n=48 |
| Best response, n (%) |  |  |  |
| Partial response | 5 (10.42) | 5 (10.42) | 5 (10.42) |
| Stable disease | 42 (87.5) | 37 (77.08) | 47 (97.92) |
| Progressive disease | 1 (2.08) | 6 (12.50) | 0 |
| ORR, (%) | 5 (10.42) | 5 (10.42) | 5 (10.42) |
| DOR (months), median (95% CI) | NR (NR-NR) | NR (NR-NR) | NR (NR-NR) |
| **Breast cancer** | n=48 | n=48 | n=48 |
| Best response, n (%) |  |  |  |
| Partial response | 6 (12.50) | 9 (18.75) | 6 (12.50) |
| Stable disease | 41 (85.42) | 35 (72.92) | 48 (100) |
| Progressive disease | 1 (2.08) | 4 (8.33) | 0 |
| ORR, (%) | 6 (12.50) | 9 (18.75) | 6 (12.50) |
| DOR (months), median (95% CI) | NR (NR-NR) | NR (NR-NR) | NR (NR-NR) |

ORR, objective response rate; DOR, duration of response; CI, confidence interval; NR, not reached

**Supplementary Table 6** AEs in subgroups by tumor type

| **Event, n (%)** | **Lung cancer**  **(n=141)** | | **Gastrointestinal cancer**  **(n=238)** | | **Gynecological cancer**  **(n=48)** | | **Breast cancer**  **(n=48)** | |
| --- | --- | --- | --- | --- | --- | --- | --- | --- |
|  | **Any grade** | **Grade ≥3** | **Any grade** | **Grade ≥3** | **Any grade** | **Grade ≥3** | **Any grade** | **Grade ≥3** |
| Any AE | 38 (26.95) | 3 (2.13) | 30 (12.61) | 3 (1.26) | 14 (29.17) | 2 (4.17) | 11 (22.92) | 0 |
| AE leading to dose reduction or interruption of anlotinib | 7 (4.96) | 0 | 5 (2.10) | 0 | 2 (4.17) | 0 | 2 (4.17) | 0 |
| AE leading to anlotinib discontinuation | 0 | 0 | 3 (1.26) | 1 (0.42) | 0 | 0 | 0 | 0 |
| AE leading to death | 0 | 0 | 0 | 0 | 0 | 0 | 0 | 0 |
| Hematological toxicities | 27 (19.15) | 3 (2.13) | 23 (9.66) | 3 (1.26) | 10 (20.83) | 2 (4.17) | 7 (14.58) | 0 |
| Gastrointestinal toxicities | 7 (4.96) | 0 | 4 (1.68) | 0 | 4 (8.33) | 0 | 2 (4.17) | 0 |
| Pain | 3 (2.13) | 0 | 2 (0.84) | 0 | 0 | 0 | 2 (4.17) | 0 |
| Fatigue | 1 (0.71) | 0 | 0 | 0 | 0 | 0 | 0 | 0 |

AE, adverse event
